# Complexity, Training Paradigm Design, and the Contribution of Memory Subsystems to Grammar Learning

**DOI:** 10.1371/journal.pone.0158812

**Published:** 2016-07-08

**Authors:** Mark Antoniou, Marc Ettlinger, Patrick C. M. Wong

**Affiliations:** 1 The MARCS Institute for Brain, Behaviour and Development, Western Sydney University, Sydney, Australia; 2 Department of Veterans Affairs Northern California Heath Care System, Davis, California, United States of America; 3 Department of Linguistics and Modern Languages, The Chinese University of Hong Kong, Shatin, Hong Kong SAR; 4 Brain and Mind Institute, The Chinese University of Hong Kong, Shatin, N.T., Hong Kong SAR; Waseda University, JAPAN

## Abstract

Although there is variability in nonnative grammar learning outcomes, the contributions of training paradigm design and memory subsystems are not well understood. To examine this, we presented learners with an artificial grammar that formed words via simple and complex morphophonological rules. Across three experiments, we manipulated training paradigm design and measured subjects' declarative, procedural, and working memory subsystems. Experiment 1 demonstrated that passive, exposure-based training boosted learning of both simple and complex grammatical rules, relative to no training. Additionally, procedural memory correlated with simple rule learning, whereas declarative memory correlated with complex rule learning. Experiment 2 showed that presenting corrective feedback during the test phase did not improve learning. Experiment 3 revealed that structuring the order of training so that subjects are first exposed to the simple rule and then the complex improved learning. The cumulative findings shed light on the contributions of grammatical complexity, training paradigm design, and domain-general memory subsystems in determining grammar learning success.

## Introduction

Second language (L2) learning is characterized by great variability in learning outcomes, and there is increasing interest in the contribution made by memory subsystems to L2 learning success [1,2]. Research has shed light on the contribution made by memory subsystems in the learning of consonants [3], vowels [4], lexical tones [5], vocabulary [6], and overall language ability [7], but much remains unknown about their role in grammar learning (but see [8–15]). This is somewhat surprising because mastery of grammar is a necessary component of L2 learning that distinguishes proficient from non-proficient L2 speakers [16], and poses particular difficulty for L2 learners [17]. Furthermore, while there is a growing body of work that examines the effectiveness of different training methods, there have been few systematic examinations of the relationship between training methods and memory subsystems [18]. The purpose of the present study is to conduct an exploratory analysis of the role of memory subsystems in grammar learning. We seek to determine if known factors such as the ordering of the complexity of training and the presentation of trial-by-trial feedback contribute to grammar learning success, and how these paradigms differentially recruit memory subsystems. Our ultimate goal is to lay the foundations for future studies to proactively tailor training based on individual cognitive profiles with the aim of maximizing grammar learning outcomes.

Grammar refers to the rules governing how linguistic units may combine in a given language, including how phonemes are combined (phonology), how words are created (morphology), and how words combine to form sentences (syntax). Non-native learners often experience difficulty in learning these grammatical rules [19]. Grammar learning depends on the process of abstracting patterns from input [20], but to date, little work has been conducted on variability in grammar learning for L2 learners. There is, however, evidence linking domain general cognitive abilities (e.g., auditory working memory, and declarative and procedural memory) and their associated brain structures to grammar learning. Thus, we might expect L2 grammar learning to vary across individuals as a function of these memory subsystems.

Working memory capacity has long been associated with L2 learning success. Baddeley et al. [21] defines auditory working memory as a phonological loop, or buffer, that mediates between auditory input and complex higher-order learning and language abilities. Greater working memory availability would improve language learning by virtue of allowing the learner to incorporate a larger amount of input into the learning process [22]. Working memory may facilitate learning by allowing relevant information to be actively attended to during processing [23]. Empirical studies support the importance of working memory in language learning (for reviews see [2,7,24]). Working memory has been shown to play an important role in a number of language skills, including reading comprehension [25], sentence comprehension [26], resolving lexical ambiguity [27,28], modifying output in the L2 [29], acquiring L2 vocabulary knowledge [30,31], and grammar learning [32].

Additionally, Ullman's [33,34] Declarative/Procedural model specifies the roles of declarative and procedural memory in language learning and use. Procedural memory underlies both motor and cognitive skill and habit learning, and is considered to be a type of implicit memory, associated with acquiring sequences [35]. Declarative memory comprises knowledge about facts and events related to the world or knowledge of events that one has experienced [36]. Simple grammatical processes are sequence-oriented, and therefore procedural, in nature. Thus, it would be reasonable to expect procedural memory to correlate with grammar learning. In contrast, the lexicon relies on declarative memory, which is specialized for arbitrary associations. In Ullman's model, the role of working memory is to allow for maintenance and structuring of rule-governed patterns (in service of procedural memory), and to manipulate selected lexical items (in service of declarative memory). However, when it comes to L2 learning, there is evidence that the relationships between the different memory systems and language become more complex. The L2 lexicon will rely on declarative memory (as was the case for the native language), but the L2 grammar, unlike the grammar of the native language, will draw on *both* declarative and procedural memory. At low L2 grammar proficiency, there is a greater reliance on declarative memory, whereas as L2 proficiency increases, the L2 grammar will rely more on procedural memory (as does the native language grammar), and working memory demands will be reduced. From a cognitive perspective, declarative memory will influence the initial stages of L2 grammar learning and procedural memory may determine ultimate attainment.

There is a growing number of studies that are investigating the contribution of declarative and procedural memory subsystems to grammar learning success [37,38]. Morgan-Short et al. [9] familiarized subjects with an artificial language and then had them complete a grammaticality judgement task in which some sentences violated word order. At early stages of acquisition (after two training sessions), grammar learning correlated with declarative, but not procedural, memory. At later stages of acquisition (after six sessions), grammar learning correlated with procedural memory, and no longer with declarative. The findings lend support to the Declarative/Procedural view [33,34] that declarative and procedural memory predict L2 grammatical development at the early and late stages of acquisition, respectively. Recently, Ettlinger et al. [8] examined the acquisition of a morphophonological grammar and how this relates to procedural, declarative, and working memory. There was considerable variation in grammar learning outcomes across subjects, but importantly, this was accounted for by declarative and procedural memory, whereas working memory did not correlate. A role for the domain-general memory system in language learning is further supported by neuroimaging evidence. Neuroimaging and lesion studies have identified a shared neural substrate consisting of a frontostriatal network incorporating Broca's area and the basal ganglia, linking procedural memory and grammar [39–41].

Given the posited roles of procedural, declarative, and working memory in L2 grammar learning, these domain-general cognitive systems may be of particular importance when assigning individuals to different types of training methods. External factors, such as training parameters, may differentially recruit memory systems. For example, one interesting contribution of Ettlinger et al. [8] is the finding that learners differed in their grammar learning success depending on whether the structure being learned was grammatically simple or complex. Learners encountered more difficulty in learning the complex morphophonological pattern than the simple one. However, acquisition of the complex pattern entailed acquisition of the simple pattern for some learners. This finding was not consistent with the well-established view that simple linguistic structures should be learned first before moving on to more advanced, complex structures [42]. For example, scaffolding in learning has been a fundamental teaching principle, according to which students incrementally extend their abilities and level of understanding [43–45]. Compatibly, connectionist modelling of language development has advocated that starting small will lead to superior learning [46–48], and this has also been demonstrated for several aspects of grammar learning [49–53]. The Ettlinger et al. [8] finding is, however, consistent with research on childhood language development and treatment of communication disorders which suggests that use of complex language material in training leads to improved language learning and treatment outcomes. For example, Gierut [54] has shown that learning phonologically complex segments leads to greater improvements and generalization in children with phonological delays. Specifically, training children to produce more complex (i.e., marked) structures such as affricates results in learning of simpler (i.e., unmarked) but related structures such as fricatives. Similarly, training clusters generalizes to singletons, and training clusters with greater sonority (e.g., /kw/) affects learning of clusters with less sonority (e.g., /bl/). Importantly, for all of the above cases, training of simpler segments did not result in gains for complex segments. Kiran [55] provides converging evidence from the lexical-semantic domain. Training of complex, atypical items within a semantic category (e.g., a *penguin* as an example of the category *birds*) boosted access of simpler, typical items in that category, whereas training of simpler items did not yield benefits for complex items. Thompson and Shapiro [56] highlight the benefits of training complex sentence structures in agrammatic aphasics. Training production and comprehension of complex sentences generalized to simpler sentence structures with the same movement, whereas training simple sentences did not generalize to complex ones. The above findings converge to suggest that greater language learning outcomes may be achieved when complex language material is used in training, rather than when simple material is used. Thus, we might expect similar benefits to be observed in the learning of grammar.

An additional factor that contributes to learning is the presentation of corrective feedback. Feedback refers to information provided to learners that verifies the occurrence of learning [57]. Learning with trial-by-trial feedback is considered to be easier than without, and improves the speed of learning as well as overall performance [58]. However, for some tasks, feedback leads to a decrement in performance [59]. Moreover, neuroimaging studies have shown that the frontostriatal network plays a crucial role in feedback-based learning [60,61]. Given that the frontostriatal network is linked to procedural memory [62], we might expect subjects with greater procedural memory availability to benefit most from feedback. It has also been suggested that presenting feedback taxes working memory [63], and therefore, learners with greater working memory capacity will benefit most from feedback [64,65]. One possible explanation is that working memory affects what information learners attend to when feedback is presented [7,29]. With regard to language training specifically, presenting feedback leads to better learning of speech sounds [66], and is generally considered to improve L2 grammar learning outcomes [67]. It is not clear how feedback might interact with the complexity of the training material, although individuals with greater procedural memory and working memory should be most likely to benefit.

In sum, past research suggests that there exists a link between memory subsystems (working, declarative, and procedural) and L2 grammar learning outcomes, although the nature and mechanism of these relationships is still unclear. Crucially, there is no clear way to translate the reviewed findings on the relationship between working memory, declarative, and procedural memory and L2 learning into improved second language pedagogy. To address this, in the present study, we examined how working memory, and declarative and procedural memory subsystems relate to different training methods of grammar learning. Specifically, we manipulated whether feedback was incorporated, how it was incorporated, and whether item ordering affects acquisition, to better understand the role that memory subsystems play in L2 grammar learning. The results may assist practitioners to pre-identify what training methods work best with which language learners based on pretesting of these memory subsystems. That is, beyond extant research that predicts L2 learning ability based on working memory, we may be able to identify which language learners will learn best with different language learning methods incorporating different types of feedback and item ordering.

We exposed subjects to the same artificial language used in Ettlinger et al. [8], containing both simple and complex grammatical processes of word-formation. Half of the words were formed using a simple grammatical pattern, and the other half were formed via a complex pattern. Following training, subjects were asked to generalize these newly learned grammatical processes to new words. The training conditions were manipulated across three experiments. In Experiment 1, the contribution of training was established by comparing grammar learning following passive, exposure-based training and test versus a condition in which training was not administered and subjects were required to learn during the test phase based on feedback alone. In Experiment 2, the contribution of feedback was assessed by comparing learning when feedback was or was not provided during test. In Experiment 3, we examined the contribution of the ordering of training by first presenting either the simple or complex grammar to assess the robustness of generalization and whether learning the complex pattern automatically entails the simple one.

## Experiment 1: The Influence of Passive, Exposure-Based Training on Grammar Learning

The present series of experiments seeks to address key issues in cognitive science concerning the role of domain-general memory subsystems in grammar learning. Before examining the contribution of feedback (Experiment 2) or ordering of complexity in training (Experiment 3), it is first necessary to demonstrate that memory subsystems play a role in the learning processes that occur during training.

For instance, it is well known in the field of artificial grammar learning that is possible for subjects to perform above chance without having learned the grammatical rules during training, either because of biases in the stimuli or because of potential learning and reasoning strategies used during test. To address this, we asked a test-only control group to complete the same artificial language tests but without having first completed training. Crucially, both groups completed a battery of cognitive tests to determine if domain-general memory subsystems relate to the initial training and not the test-specific strategies and reasoning in which subjects may potentially engage.

The aim of Experiment 1 was to test the contribution of passive, exposure-based training to grammar learning outcomes. It was hypothesized that learning outcomes would be greater when training preceded test relative to the test-alone condition (with no training). Further, if as we expect memory abilities correlate with learning of the simple and complex grammatical rules, then we would expect these relationships to be diminished in the test-alone controls.

### Method

#### Ethics Statement

This study was performed in strict accordance with an approved protocol. Subjects provided informed written consent in accordance with the Institutional Review Board and all experimental procedures were approved by the Northwestern University Institutional Review Board.

#### Subjects

In total across Experiments 1–3, one hundred and twenty-two native English speakers who were students at Northwestern University took part in the study. Some subjects reported that they possessed experience with another language (Arabic *n* = 1, Bulgarian *n* = 1, French *n* = 8, German *n* = 2, Hebrew *n* = 3, Mandarin *n* = 3, and Spanish *n* = 26), but none considered themselves to be native speakers (self-ratings ≤ 4 out of 7), and none of these languages have structures similar to those present in our study. Subjects gave informed consent and were monetarily compensated for their time. All were free of neurological deficits and passed a pure tone audiological screening at 25 dB HL at 500, 1,000, 2,000, and 4,000 Hz.

In Experiment 1, to assess the contribution of passive, exposure-based training to grammar learning, 36 subjects completed the training followed by the test phase with feedback (baseline group) (*M*_age_ = 23.1; *SD =* 2.3; 21 females), and 25 subjects completed only the test phase with feedback, but no training (test-only group) (*M*_age_ = 21.9; *SD* = 2.3; 17 females). As is shown in Table 1, the groups were matched for declarative memory, procedural memory, and working memory.

**Table 1:** Group sizes, mean ages, and memory measures for baseline and test-only groups in Experiment 1. Bottom row shows p-values from t-tests confirming that the groups were matched on each memory measure.

Group	n	*M* Age (*SD*)	Dec M	Proc M	WM
Baseline	36	23.1 (2.3)	.602	.505	.766
Test-only	25	21.9 (3.0)	.551	.500	.700
*p*-value			.45	.95	.19

#### Stimulus materials

The artificial language was comprised of 30 noun-stems and two affixes that combined to form 120 words. The nouns were consonant-vowel-consonant monosyllables and represented 30 different animals (e.g., [pag] represented dog). The prefix [ka-] signalled the diminutive (e.g., doggy), and the suffix [-il] signalled the plural (e.g., dogs). The phonemic inventory consisted of American English consonants and the three vowels [a, e, i]. Each vowel was used in 10 word noun-stems (e.g., pag, zek, kij represented dog, sheep, rooster, respectively).

The grammar of the artificial language had two types of word formation rules, one simple and the other complex, and is modeled after grammar rules in a natural language, Shimakonde. In Shimakonde, plural and diminutive affixes combine with noun stems to create new words (Liphola 2001; Ettlinger, 2008). In our artificial grammar, the simple rule involved concatenating noun-stems with the suffix [-il] and/or prefix [ka-] (e.g., pag, pagil, kapag represent dog, dogs, doggy, respectively). The complex rule required concatenation and changing the vowels of both the stem and the affix, reflecting two phonological processes that do not occur in English. The first process is vowel harmony, which changes the vowel of the suffix to match that in the noun-stem (e.g., the plural of *pag* is *pagil* (simple) but *zek* is *zekel*). The second process is reduction, which changes noun stem vowels to match the prefix [ka-] (e.g., the diminutive of *zek* is *kazak*). These two processes may combine to form complex words (e.g., *kazakel* meaning many little sheep) and these contrast with simple words (e.g., *kapagil* meaning many little doggies).

For the experiment, each word was paired with a picture of a common animal (for a full stimulus list see [8]). A native English speaker produced the words at a normal rate with English prosody and phonology. Recordings were made using a Shure SM58 microphone and were digitized in Praat (16-bit, 22.05 kHz).

#### Procedure

Subjects were instructed that they would be tested on a new language. No instructions were given concerning the rules of the language or that there were any rules to learn. For subjects that underwent training, they were presented with picture-spoken word pairings (e.g., a picture of a dog was shown and [pag] was heard). Twelve nouns were presented in all four forms in the following order: singular, diminutive, plural, and diminutive plural. Half of the nouns were simple, and the other half were complex, and their presentation order was randomized. Each exposure block was repeated four times, resulting in 192 training trials in total (12 nouns × 4 forms × 4 repetitions = 192 exposures). Each noun was presented onscreen as a picture for 3 s. The spoken word naming the picture was played 500 ms after the picture had appeared. At the end of the 3 s exposure, the picture disappeared and the screen remained blank for 500 ms before the next noun was presented. Upon completion of training, subjects were given a short break before moving on to the test phase.

Subjects were tested on their ability to apply the newly learned grammatical rules to novel words in a modified wug test [68]. A wug test requires subjects to modify a previously unencountered word from its singular form (e.g., wug) to produce it in a different form (e.g., plural form = wugs). In our version of the wug test, subjects were exposed to a new picture (out of 18 unencountered nouns) for 1.5 s and 500 ms after the new picture appeared, the spoken word naming the singular form of the picture was played. The picture then disappeared and the screen remained blank for 1 s. Subjects were then presented with a picture of the same noun but in a different form (either plural, diminutive, or diminutive plural). For example, if the subject had been exposed to a lion, they might now be presented with a picture of many small lions. Two spoken words were provided as response options and subjects were required to select the correct word by pressing one of two buttons on a response box within a 5 s response time limit. The plural, diminutive, and diminutive plural forms of each of the 18 unencountered nouns were tested in random order, with singular forms always used as the prompt. This resulted in a total of 54 test trials (18 nouns × 3 forms = 54 test trials). Foil responses were always the alternative affix (i.e., [-el] vs. [-il] for plurals, [a] vs. [e] for diminutives, and [-el] vs. [-il] for diminutive plurals). The order of the foil and correct words as response options 1 and 2 was counterbalanced. After a response, feedback was provided indicating whether they had made a correct or incorrect response. For incorrect trials, subjects were also played back the correct answer. Stimulus presentation was controlled by a computer running E-Prime (Psychology Software Tools, Pittsburgh, PA). Auditory stimuli were presented at about 72 dB SPL via Sennheiser HD 280 PRO headphones and visual stimuli were presented on a computer monitor. Responses were recorded using a low-latency button box.

#### Cognitive tests

A cognitive test battery was administered to measure subjects' procedural, declarative and working memory subsystems. The tests were selected because they have been used in closely related past work [8,9].

Procedural memory was assessed using a computerized version of the Tower of London (TOL) test [69]. In the TOL, subjects are presented with an arrangement of balls stacked on pegs and are required to move balls one at a time in order to arrive at a goal arrangement. As the test progresses, trials become more difficult, such that the minimum number of moves required increases. The same start-goal sequences are repeated later in the test, and any improvement in performance is taken as a measure of procedural learning [70,71]. Each participant's score on the second repetition of sequences was normalized relative to the rest of the group.

Declarative memory was assessed using the Visual-Auditory Learning subtest of the Woodcock-Johnson III (WJ-III) Tests of Cognitive Abilities [72]. Subjects are required to learn to associate new visual symbols with orally presented words. Sequences of the newly learned symbols are then presented and the subject is required to 'read' them aloud. The score is standardized such that the population mean corresponds to a score of 100.

Working memory was assessed using the Auditory Working Memory subtest of the WJ-III. In this subtest, subjects are required to form categories of words and digits while retaining the appropriate sequence of the items. A series of intermixed digits and words are presented via audio recorded stimuli (e.g., “dog, 1, shoe, 8, 2, apple”). The subjects' task is to first repeat the words in sequential order (e.g., dog, shoe, apple) and then the digits in order (e.g., 1, 8, 2). The score is standardized such that the population mean corresponds to a score of 100.

### Results and Discussion

**Fig 1:**
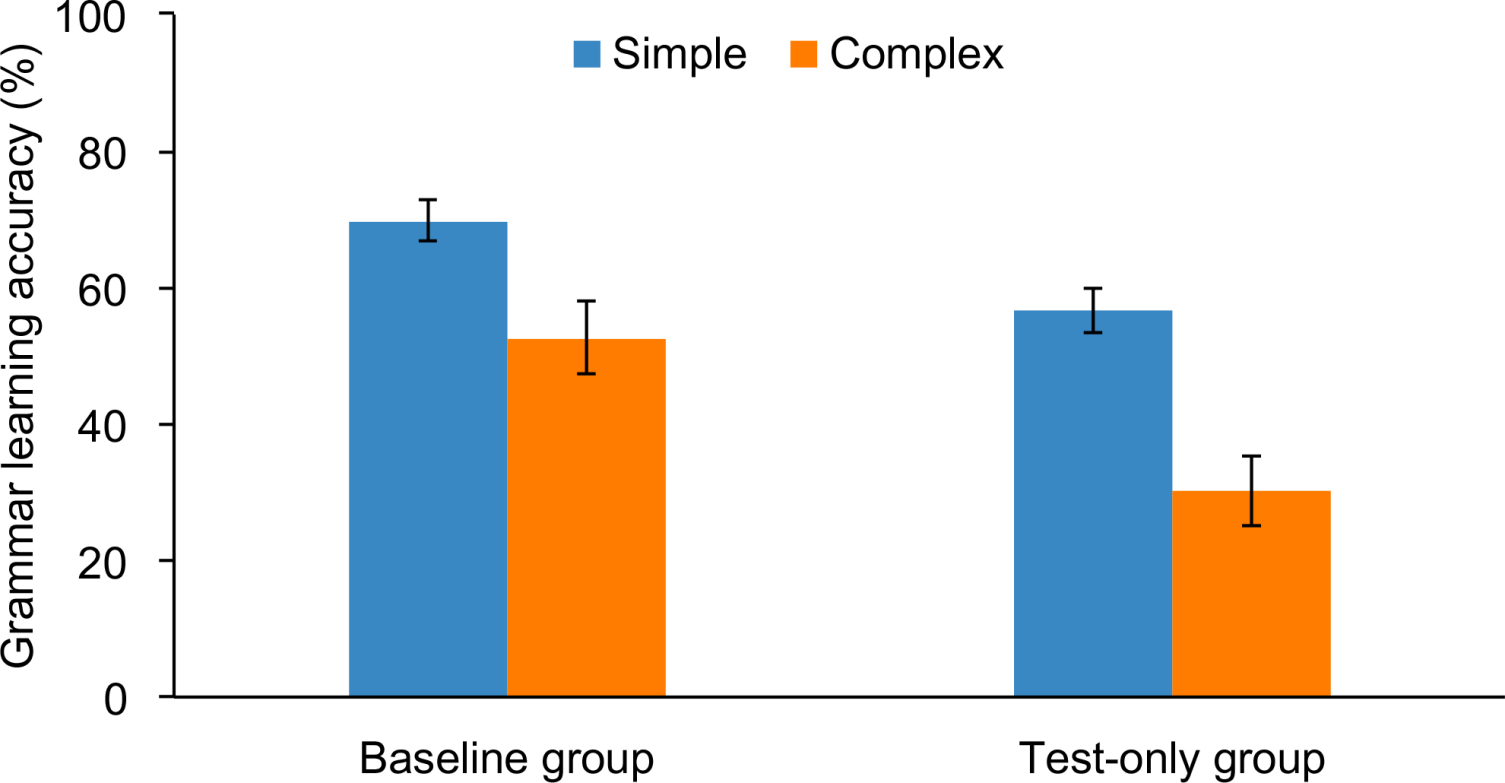
Grammar learning accuracy (%) of simple and complex grammar rules by subjects who underwent passive, exposure-based training + test (baseline group) versus those who immediately completed the test without first undergoing training (test-only group). Errors bars depict the standard error of the mean.

We examined the contribution of training to grammar learning by conducting a 2 × (2) ANOVA with the between-subjects factor of group (baseline vs. test-only) and a within-subjects factor of grammar (simple vs. complex). The S1 File lists subject codes for Experiment 1, their grammar learning scores, and measures of declarative, procedural, and working memory. A main effect of group, *F*(1, 59) = 15.3, *p* < .001, $\eta_{p}^{2}$ = .206, revealed that overall the baseline group (61.2%) outperformed the test-only group (43.4%). A main effect of grammar, *F*(1, 59) = 23.3, *p* < .001, $\eta_{p}^{2}$ = .283, revealed that overall the simple grammar (64.3%) was easier to learn than the complex (42.5%). There was no significant interaction, *p* = .314. As shown in Fig 1, these findings suggest that passive exposure to a nonnative language boosts learning of both simple and complex grammatical rules. The only learning effect that did not exceed chance level was learning of the complex rule by the test-only group.

**Fig 2:**
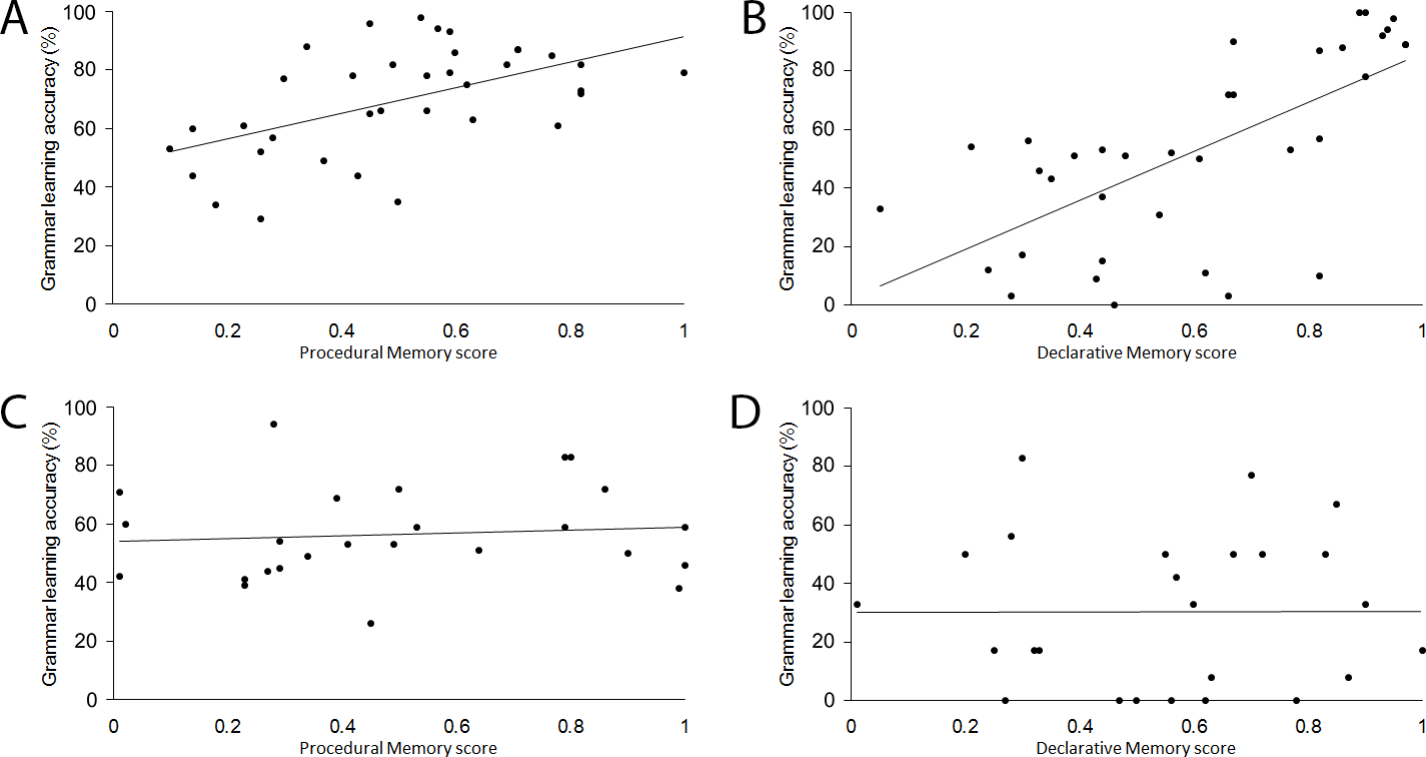
Scatterplots depicting correlations for baseline group between (A) procedural memory and simple grammar learning, and (B) declarative memory and complex grammar learning. For test-only group, procedural and declarative memory did not correlate with simple or complex grammar learning (C and D, respectively).

To test if domain-general measures of memory subsystems are linked to grammar learning, a series of stepwise multiple regression analyses were conducted for both simple and complex grammar learning, examining the unique contributions made by measures of procedural, declarative, and working memory. As is shown in Table 2, for subjects in the baseline condition, a significant correlation was found between learning of the simple grammar and procedural memory, but not declarative memory or working memory, and a significant correlation was also found between learning of the complex grammar and declarative memory, but not procedural memory or working memory. The test-only group did not show the same patterns of correlations (see Fig 2), suggesting that these memory systems are recruited during learning that occurs prior to the test phase. For the test-only group, the only significant correlation was a negative correlation between declarative memory and learning of the simple grammar.

**Table 2:** Pearson correlations between the memory measures and learning of the simple and complex grammatical patterns by the baseline and test-only groups.

Group	Variable	Dec M	Proc M	WM
Baseline	Proc M	.268		
	WM	.288*	.106	
	Simple	.159	.531***	.106
	Complex	.669***	.140	–.030
Test-only	Proc M	.113		
	WM	.077	–.068	
	Simple	–.430*	.096	.045
	Complex	.001	.004	.167

*** <. 001

* < .05.

For the baseline group, the multiple regressions revealed that for the simple grammar the only significant predictor was procedural memory, *B* = .437, β = .531, *R*^*2*^ = .282, adjusted *R*^*2*^ = .261, *p* = .001, and for the complex grammar the only significant predictor was declarative memory, *B* = .837, β = .669, *R*^*2*^ = .448, adjusted *R*^*2*^ = .431, *p* < .001. For the test-only group, declarative memory was the only significant predictor for the simple grammar, *B* = –.273, β = –.430, *R*^*2*^ = .185, adjusted *R*^*2*^ = .150, *p* = .032, and there were no significant predictors for the complex grammar. It appears that the complex rule was too difficult for memory effects to emerge in the test-only condition.

The findings of Experiment 1 demonstrate that (a) exposure-based learning prior to test leads to superior grammar learning than test alone, and (b) procedural and declarative memory abilities correlate with learning of simple and complex grammatical patterns, respectively. Crucially, these relationships between memory subsystems and grammar learning were only observed for the baseline group and not the test-only group, which suggests that these memory systems are implicated in grammar learning as we hypothesize, and rule out alternative explanations such as that subjects improvements were due to their ability to detect biases in the stimuli or because of potential learning and reasoning during test.

## Experiment 2: The Contribution of Feedback to Grammar Learning

Having demonstrated that exposure-based training leads to superior learning of grammatical rules than learning that occurs solely due to feedback during test, we next examined the contribution of feedback during the test phase. Feedback is thought to improve grammar learning [67]. Thus, one might expect that the presentation of feedback during the test phase would boost learning outcomes relative to a condition in which no feedback is provided during testing. Furthermore, the frontostriatal network plays a crucial role in feedback-based learning [60,61] and has also been linked to procedural memory [62]. It has also been suggested that presenting feedback taxes working memory [63], and learners with greater working memory capacity will benefit most from feedback [64,65]. Therefore, those individuals with better procedural memory and working memory might benefit most from feedback. If the presentation of feedback during test does not significantly boost learning outcomes, this would suggest that most learning occurs during the exposure phase, rather than during test.

### Method

#### Subjects

In order to assess the contribution of feedback presented during the test phase to grammar learning outcomes, 25 subjects completed the training followed by a test phase without feedback (no-feedback group) (M_age_ = 21.8; SD = 1.3; 13 females), and their performance was compared to the 36 baseline group subjects who completed the training and test with feedback (see *Experiment 1*). As is shown in Table 3, the groups were matched for declarative memory, procedural memory, and working memory.

**Table 3:** Group sizes, mean ages, and memory measures for the baseline and no-feedback groups in Experiment 2. Bottom row shows p-values from t-tests confirming that the groups were matched on each memory measure.

Group	n	*M* Age (*SD*)	Dec M	Proc M	WM
Baseline	36	23.1 (2.3)	.602	.505	.766
No-feedback	25	21.8 (1.3)	.532	.552	.687
*p*-value			.28	.44	.18

#### Stimulus materials

The same artificial language was used as in Experiment 1.

#### Procedure

Subjects in the baseline experimental condition (who completed training and then the test with feedback) were compared with subjects who did not receive feedback during testing. Subjects in the no-feedback condition completed the same training as those in the baseline group, but no feedback was provided during the test phase.

### Results and Discussion

**Fig 3:**
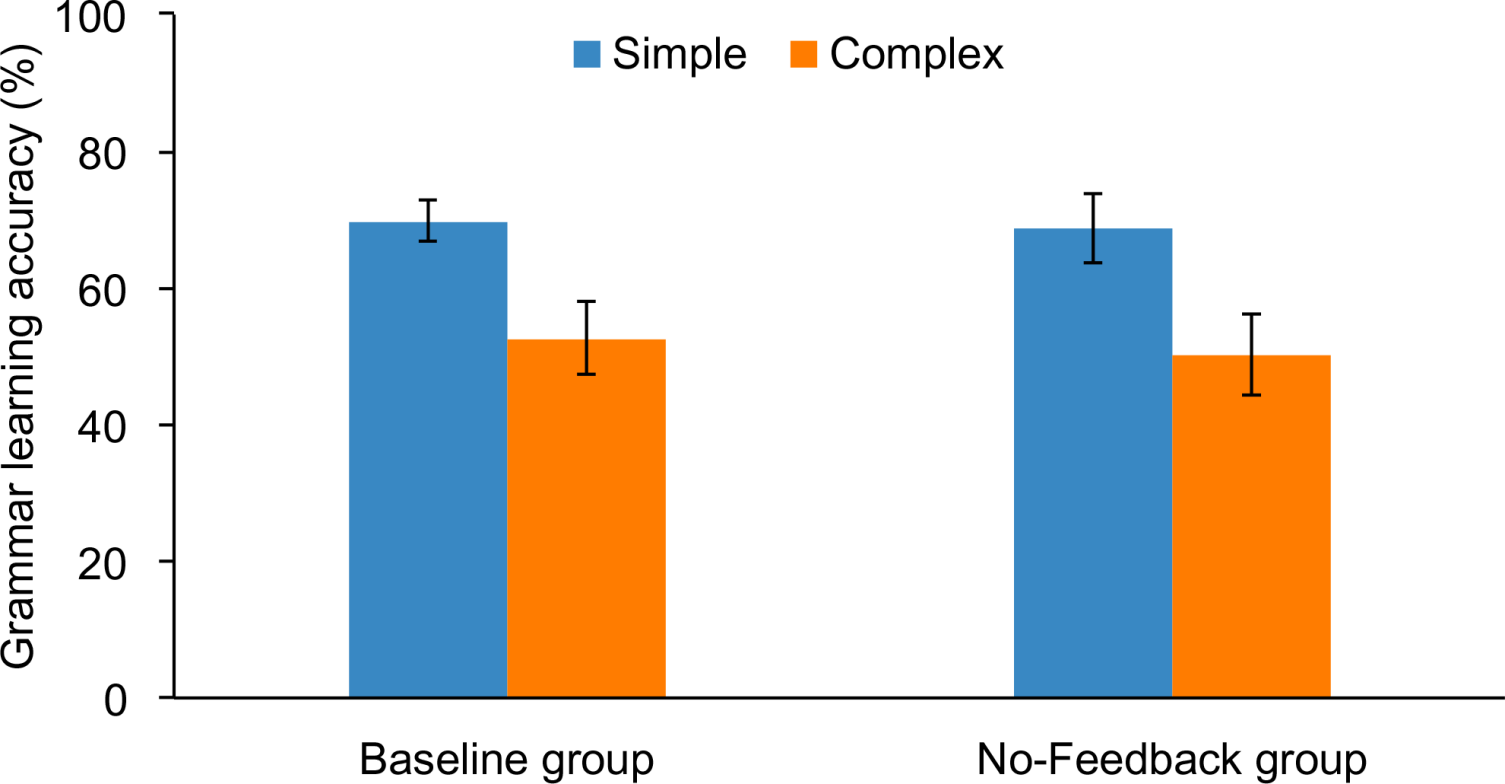
Grammar learning accuracy (%) of simple and complex grammar rules by subjects who were tested with or without feedback (baseline vs. no-feedback groups, respectively). Error bars depict standard errror of the mean.

We tested the contribution of feedback to grammar learning by comparing the baseline condition (which received feedback during the test phase) with the no-feedback condition. We conducted a 2 × (2) ANOVA with the between-subjects factor of feedback (feedback vs. no feedback) and a within-subjects factor of grammar (simple vs. complex). The S2 File lists subject codes for Experiment 2, their grammar learning scores, and measures of declarative, procedural, and working memory. A main effect of grammar, *F*(1, 59) = 12.2, *p* = .001, $\eta_{p}^{2}$ = .172, revealed that overall the simple grammar (69.3%) was easier to learn than the complex (51.6%), as is shown in Fig 3. There was no significant main effect of feedback, *p* = .719, or interaction, *p* = .877.

**Fig 4:**
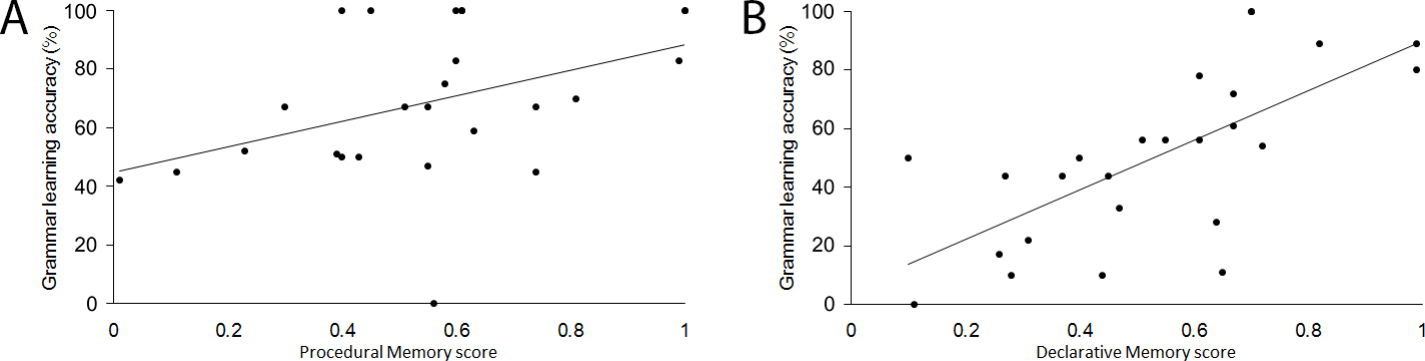
Scatterplots depicting correlations for no-feedback group between (A) procedural memory and simple grammar learning, and (B) declarative memory and complex grammar learning.

Correlations between memory subsystems and grammar learning are shown in Table 4. For subjects in the no-feedback condition, stepwise multiple regression analyses revealed that the only significant predictor of simple grammar learning was procedural memory, *B* = .434, β = .424, *R*^*2*^ = .180, adjusted *R*^*2*^ = .144, *p* = .035, and the only significant predictor of complex grammar learning was declarative memory, *B* = .848, β =. 690, *R*^*2*^ = .476, adjusted *R*^*2*^ = .453, *p* < .001. This pattern was very similar to that which was observed for the baseline group (compare Fig 4A and 4B to Fig 2A and 2B). Recall that for baseline (i.e., with feedback) subjects declarative memory was the sole predictor of complex grammar learning, and procedural memory was the only predictor of simple grammar learning (reported in Experiment 1).

**Table 4:** Pearson correlations between the memory measures and learning of the simple and complex grammatical patterns by the baseline and no-feedback groups.

Group	Variable	Dec M	Proc M	WM
Baseline	Proc M	.268		
	WM	.288*	.106	
	Simple	.159	.531***	.106
	Complex	.669***	.140	–.030
No-feedback	Proc M	.261		
	WM	.034	.196	
	Simple	.220	.424*	.023
	Complex	.690***	.068	–.001

*** <. 001

* < .05.

## Experiment 3: Training Order Effects on Grammar Learning

Having established that passive, exposure-based training boosts learning of simple and complex grammatical patterns, and that domain-general memory subsystems differentially relate to learning of simple and complex grammatical patterns, Experiment 3 examined the effect of ordering of training. Based on our review of the literature on complexity, we developed two competing hypotheses, both of which predict that ordering of training will exert a strong effect on learning and generalization outcomes, but in opposite directions. According to the scaffolding hypothesis, mastery of the simple grammatical pattern would aid subsequent acquisition of the complex pattern, analogous to the incremental development that occurs in traditional language learning and development [43–47]. In contrast, the complexity hypothesis predicted that complex-simple training would lead to better grammar learning and generalization than the simple-complex order based on converging evidence that using complex material in language training leads to greater learning outcomes for phonology [54], semantics [55], and syntax [56].

### Method

#### Subjects

To test the effect of ordering of training, 17 subjects were trained first on the simple grammar and then on the complex grammar (simple-complex group) (M_age_ = 21.5; SD = 2.2; 9 females), and 19 subjects completed the inverse training order (complex-simple group) (M_age_ = 20.6; SD = 1.7; 12 females). As is shown in Table 5, the groups were matched for declarative memory, procedural memory, and working memory.

**Table 5:** Group sizes, mean ages, and measures of memory subsystems for Simple-complex and Complex-simple training order groups in Experiment 3. Bottom row shows p-values from t-tests confirming that the groups were matched on each memory measure.

Group	n	*M* Age (*SD*)	Dec M	Proc M	WM
Simple-complex	17	21.5 (2.2)	.565	.512	.815
Complex-simple	19	20.6 (1.7)	.584	.515	.800
*p*-value			.82	.98	.80

#### Stimulus materials

The same artificial language was used as in Experiments 1 and 2.

#### Procedure

Training was exposure-based, as in Experiments 1 and 2. Subjects in the simple-complex condition first completed a block of simple grammar training followed by a complex grammar training block. Stimuli within each block were randomized. The four forms based on the same stem were presented together, but noun stems were randomized such that those affected similarly by vowel harmony/reduction were not presented sequentially. After both blocks were completed, the experiment moved on to the test phase, during which feedback was provided. For incorrect trials, subjects were also played back the correct answer. Within the test phase, simple and complex trials were randomized. The complex-simple condition followed an identical training and test protocol with the critical difference being that the order of the training blocks was inverted: Subjects were first presented with the complex training block followed by the simple training block.

### Results and Discussion

**Fig 5:**
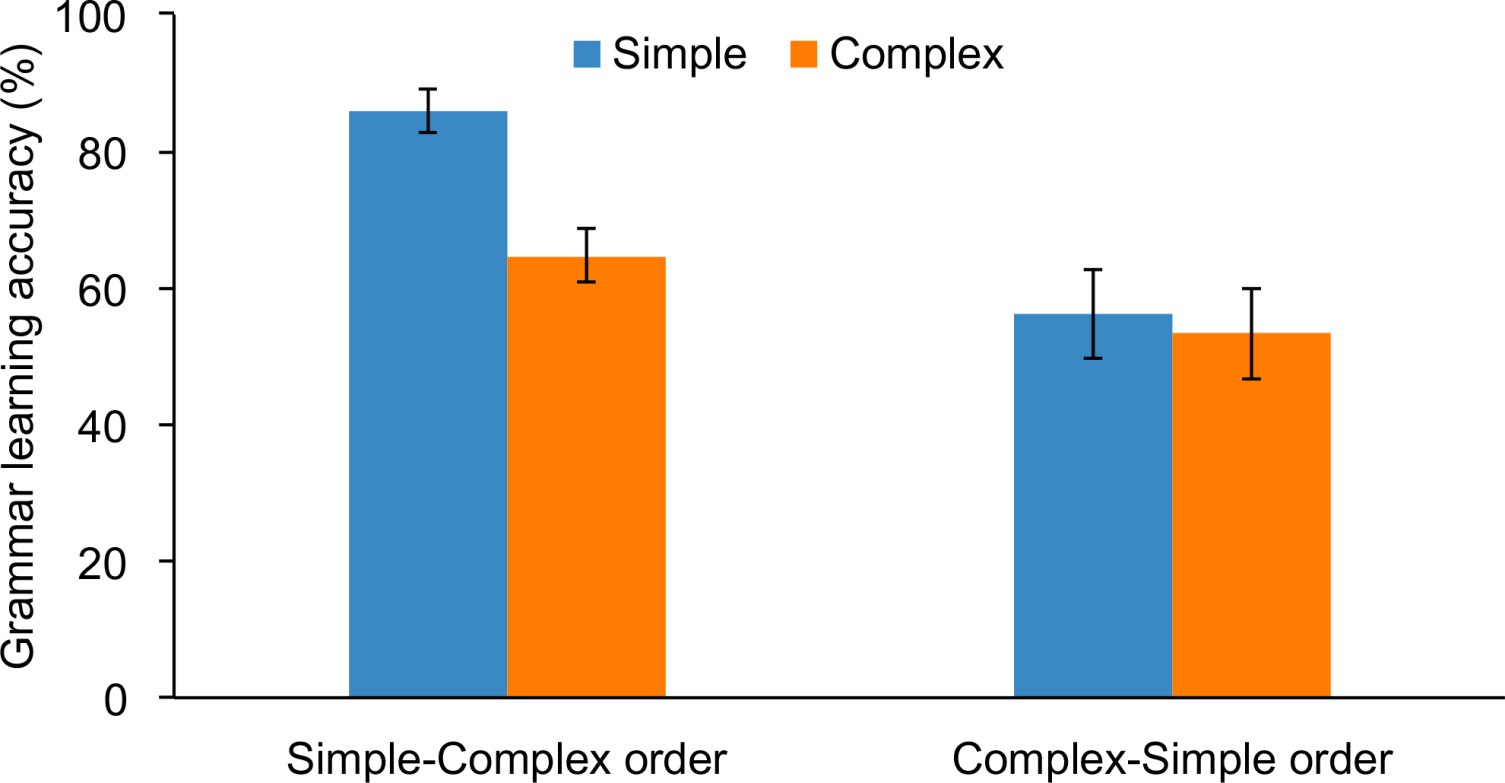
Grammar learning accuracy (%) of simple and complex grammar rules by subjects who underwent simple-complex or complex-simple training orders. Error bars depict standard error of the mean.

In order to examine the effect of the ordering of the training blocks on grammar learning we conducted a 2 × (2) ANOVA with the between-subjects factor of training order (simple-complex vs. complex-simple) and the within-subjects factor of grammar (simple vs. complex). The S3 File lists subject codes for Experiment 3, their grammar learning scores, and measures of declarative, procedural, and working memory. A main effect of training order, *F*(1, 34) = 8.2, *p* = .007, $\eta_{p}^{2}$ = .195, revealed that the simple-complex training order (75.4%) resulted in better grammar learning than the complex-simple order (54.7%). Thus, for grammar learning, ordering training so that the complex material is taught first does not seem to lead to superior learning. A main effect of grammar, *F*(1, 34) = 17.8, *p* < .001, $\eta_{p}^{2}$ = .344, revealed that, overall, the simple grammar (70.3%) resulted in better learning than the complex (58.6%). Importantly, there was a significant interaction, *F*(1, 34) = 10.0, *p* = .003, $\eta_{p}^{2}$ = .227. Bonferroni-adjusted post-hoc tests confirmed that subjects in the simple-complex training order (86.0%) outperformed those in the complex-simple order (56.2%) for the simple grammar, *t*(25.3) = 4.0, *p* < .001, whereas the two training orders did not differ on the complex grammar (64.7% vs. 53.2%), *p* = .141 (see Fig 5).

**Fig 6:**
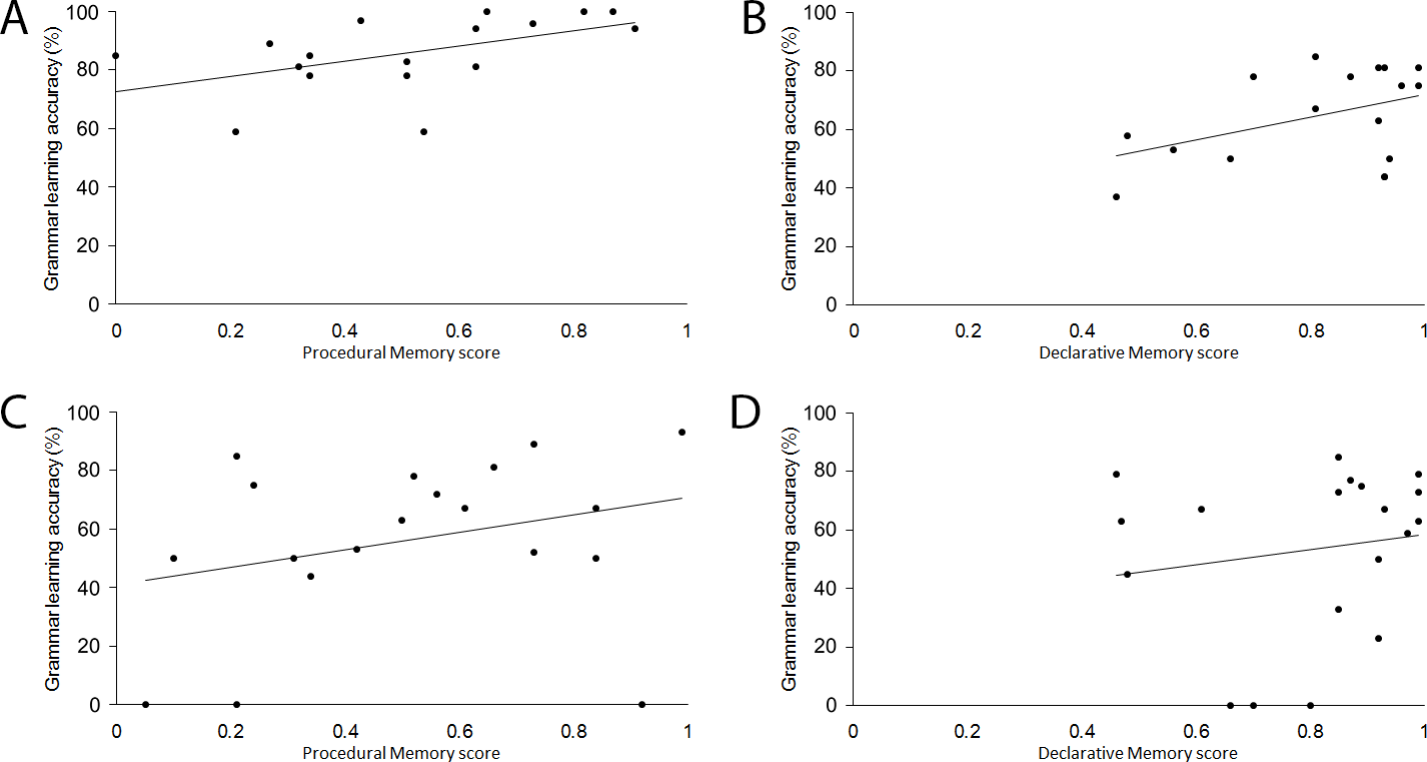
Scatterplots depicting correlations for the simple-complex training order between (A) procedural memory and simple grammar learning, and (B) declarative memory and complex grammar learning. For the complex-simple training order, procedural and declarative memory did not reliably correlate with simple or complex grammar learning (C and D, respectively).

Correlations between grammar learning and memory measures are shown in Table 6. For the simple-complex training order, procedural memory correlated with simple grammar learning, and declarative memory correlated with complex grammar learning (see Fig 6). These patterns were not observed for the complex-simple training order group for whom declarative memory positively correlated with learning of the simple rule.

**Table 6:** Pearson correlations between the memory measures and learning of the simple and complex grammatical patterns by the simple-complex and complex-simple training order groups.

Group	Variable	Dec M	Proc M	WM
Simple-complex	Proc M	–.214		
	WM	.509*	.068	
	Simple	.033	.509*	.175
	Complex	.431*	.246	.186
Complex-simple	Proc M	.305		
	WM	.093	.176	
	Simple	.407*	.293	.026
	Complex	.163	.165	–.130

* < .05.

Stepwise multiple regression analyses revealed that for the simple-complex training order, the only significant predictor of simple grammar learning was procedural memory, *B* = .260, β = .509, *R*^*2*^ = .259, adjusted *R*^*2*^ = .209, *p* = .037, and there were no significant predictors of complex grammar learning. For the complex-simple training order group, there were no significant predictors of either simple or complex grammar learning.

The findings of Experiment 3 demonstrate that ordering of complexity exerts a large effect on grammar learning outcomes. Beginning training with exposure to simple grammatical rules before moving on to complex rules leads to superior learning. Additionally, the ordering of simple and complex training differentially recruits memory subsystems.

## General Discussion

The present study is an exploratory analysis of the relationship between memory subsystems and different training parameters in morphophonological grammar learning. We examined the contribution of three factors of training paradigm design to grammar learning success, namely, the importance of passive exposure-based training (Experiment 1), the contribution of corrective feedback during the test phase (Experiment 2), and the effect of the ordering of the simple versus complex training blocks (Experiment 3). We investigated how these factors related to learning grammatical rules that were simple or complex, as well as the learners' declarative, procedural, and working memory abilities.

The findings of Experiment 1 clearly demonstrate that passive, exposure-based training makes an important contribution to grammar learning. Subjects in the baseline group (which involved training + test) achieved better learning outcomes than those in the test-only control condition. Among those who received training (baseline group), procedural memory ability was associated with positive outcomes in learning the simple grammatical rule, and declarative memory was linked with better learning of the complex grammatical rule. The results from the no-feedback group in Experiment 2 demonstrate that this relationship between memory subsystems and grammar learning persists even when feedback is not presented during the test phase. These findings are consistent with those of Ettlinger et al. [8], who found that procedural memory correlated with the learning of simple grammatical rules and declarative memory correlated with complex grammatical rules. Procedural memory supports combination in syntax [73] and the ability to track dependencies among units, more broadly [74]. Declarative memory supports word learning [75], and is crucial for early stages of L2 learning through the memorization of chunks of speech [33,34,76]. Importantly, the inclusion of training prior to test was essential in order to observe these memory subsystem relationships. Test-only subjects did not show these declarative and procedural memory effects, suggesting that procedural and declarative memory are important during the acquisition phase, while there is no evidence that they play a role during the test phase. This provides insight into the potential mechanisms involved in memory supporting language acquisition, namely that domain-general memory subsystems are differentially recruited during grammar learning. Furthermore, this finding runs counter to previous research suggesting that cognitive factors play a more important role with more complex tasks and less training [77–79]. This suggests a complex relationship between memory and language learning methodology. In the present study, higher cognitive function improved the ability to learn from data; other studies seem to suggest higher cognitive function improves the ability to extrapolate from minimal data. The amount of input may be crucial in explaining this difference between studies. Ultimately, understanding the relationship between the intricacies of memory subsystems and language learning will allow for improved language-learning pedagogy.

The data from Experiment 3 show that training order contributes to grammar learning success; the pattern observed conflicts with the complexity hypothesis and supports the scaffolding hypothesis. Subjects who were first trained on the simple grammar (simple-complex group) achieved better learning outcomes for the simple grammar than those subjects who received the complex-simple training order. Training order did not affect learning of the complex grammar. Thus, it appears that initial exposure to the complex grammar may hinder the subsequent learning of the simple grammar and does not entail, or even benefit, learning of the complex. These findings support the scaffolding hypothesis [43–45] and are consistent with past work advocating learning simple structures before moving on to more complex structures [42,46,47]. The findings differ from those of Gierut and others who have demonstrated that learning complex material aids the acquisition of simple material whether they be segments [54], semantic categories [55], or sentence structures [56]. While it might be the case that complex training leads to benefits in the acquisition of these language structures, different generalizations may apply for different types of grammar. Phonology and phonetics have a closer relationship to physiological constraints [80], syntax is more likely to have long-distance dependencies [81], semantic categories are often more easily learned hierarchically [82]; these differences and others may contribute to the way stimulus ordering affects language learning because of the nature of the generalization to be learned and quantity of quality of data used to learn the generalizations. In the present study, we have shown one case of where learning a complex grammar does not always facilitate learning a simple grammar, showing that the type of grammatical complexity matters.

Our findings additionally provide insight into how various memory subsystems contribute to learning of grammatical structures. Specifically, they suggest that procedural memory facilitates the learning of sequential information, and thus plays an important role in grammar learning (as was observed in Experiments 1, 2 and 3). This is consistent with studies showing that procedural memory supports combination in syntax [73] and the broader conception that procedural memory is linked to the ability to track dependencies [74]. Whether our observations concerning ordering and training paradigm design also generalize to other kinds of grammar learning (e.g., syntactic rather than morphophonological) awaits to be tested in future research.

Declarative memory facilitates learning of complex grammatical rules in a nonnative language, particularly during the initial stages (as was observed in Experiments 1 and 2). Ettlinger et al. [8] proposed that when completing a wug test declarative memory may store morphophonological paradigms, allowing learners to generalize the complex pattern. Their proposal is supported by our results. Consistent with this proposal, it has been suggested that declarative memory supports the learning of nonlinguistic analogical relationships [35,83] and plays an important role in learning complex, analogy-based patterns [34,84]. According to the Declarative/Procedural model [33,34], as L2 proficiency increases, the L2 grammar will come to rely on procedural memory. We did not observe such a shift for learning of the complex grammar in any of our three experiments. The language training protocol that we employed was likely too short to bring about such changes. We speculate that had we included multiple sessions of grammar training, such a shift from declarative to procedural memory may have been observed [9].

A surprising finding was that working memory did not reliably correlate with grammar learning in any of the three experiments. Previous studies have shown that working memory plays an important role in vocabulary learning [24,31], perceiving speech in noise [85], and resolving talker variability [86,87]. Martin and Ellis [32] also reported significant correlations between working memory and grammar learning, including generalization of grammatical patterns to novel utterances. The present findings have not replicated their results. Consistent with our results, however, Ettlinger et al. [8] also found that working memory did not play a role in the acquisition of simple or complex grammatical patterns. Harrington and Sawyer [22] observed a relationship between working memory and grammatical abilities in a reading task, but the methodological differences between that study and our auditorily-presented task make comparisons difficult across studies.

We also expected to observe a relationship between working memory and the ability to benefit from feedback, but this was not the case. In Experiment 2, working memory did not reliably correlate with simple or complex grammar learning, regardless of whether or not feedback was presented during the test phase. Several studies have shown that individuals with greater working memory availability benefit more from feedback [63–65]. One reason for this discrepancy may be that feedback was given during the test phase, rather than during the training phase. Future studies may adopt a trial-by-trial feedback approach to training, which may place greater demands on working memory. We leave it to future investigations to extend the present work and explore the relationships of the declarative, procedural, and working memory subsystems to grammar learning.

An important limitation of the present study is that it has not directly examined the interaction between individual differences in memory and training-related parameters on grammar learning performance. As a first step, the present results have elucidated which memory subsystems are recruited by simple versus complex grammar learning under a variety of training conditions. These findings serve as a springboard to address the interaction between individual differences in memory abilities and grammar learning in future studies. The next step is to build on this knowledge by testing a priori predictions concerning individual differences in these memory abilities in groups of subjects with high or low declarative and/or procedural memory, and examining how such groups perform on simple versus complex grammar learning.

In conclusion, grammatical complexity, training paradigm design, and domain-general memory subsystems contribute to shape grammar learning success. Passive, exposure-based training boosts the learning of both simple and complex grammatical rules. The ordering of training contributes to grammar learning such that initial exposure to simple (rather than complex) material leads to better learning. Finally, procedural memory supports the learning of simple grammatical patterns, whereas declarative memory supports the acquisition of complex patterns. By considering these training paradigm factors and taking into account individual differences in memory abilities, we may better understand how to tailor nonnative grammar learning to maximize learning outcomes.

## Supporting Information

S1 File Grammar learning data, subject codes, and measures of declarative, procedural, and working memory for Experiment 1. (SAV)

S2 File Grammar learning data, subject codes, and measures of declarative, procedural, and working memory for Experiment 2. (SAV)

S3 File Grammar learning data, subject codes, and measures of declarative, procedural, and working memory for Experiment 3. (SAV)

## References

[1] Golestani N, Zatorre RJ. Individual differences in the acquisition of second language phonology. Brain Lang. 2009;109: 55–67. 10.1016/j.bandl.2008.01.005 18295875

[2] Linck JA, Osthus P, Koeth JT, Bunting MF. Working memory and second language comprehension and production: A meta-analysis. Psychon Bull Rev. 2014;21: 861–883. 10.3758/s13423-013-0565-2 24366687

[3] Pisoni DB, Aslin RN, Perey AJ, Hennessy BL. Some effects of laboratory training on identification and discrimination of voicing contrasts in stop consonants. J Exp Psychol Hum Percept Perform. 1982;8: 297–314. 10.1037/0096-1523.8.2.297 6461723 PMC3495319

[4] Kondaurova MV, Francis AL. The relationship between native allophonic experience with vowel duration and perception of the English tense/lax vowel contrast by Spanish and Russian listeners. J Acoust Soc Am. 2008;124: 3959–3971. 10.1121/1.2999341 19206820 PMC2737250

[5] Perrachione TK, Wong PCM. Learning to recognize speakers of a non-native language: Implications for the functional organization of human auditory cortex. Neuropsychologia. 2007;45: 1899–1910. 10.1016/j.neuropsychologia.2006.11.015 17258240

[6] Kaushanskaya M, Marian V. Bilingualism reduces native-language interference during novel-word learning. J Exp Psychol Learn Mem Cogn. 2009;35: 829–835. 10.1037/a0015275 19379054

[7] Robinson P. Aptitude and second language acquisition. Annu Rev Appl Linguist. 2005;25: 46–73. 10.1017/S0267190505000036

[8] Ettlinger M, Bradlow AR, Wong PCM. Variability in the learning of complex morphophonology. Appl Psycholinguist. 2014;35: 807–831. 10.1017/S0142716412000586

[9] Morgan-Short K, Faretta-Stutenberg M, Brill-Schuetz KA, Carpenter H, Wong PCM. Declarative and procedural memory as individual differences in second language acquisition. Biling Lang Cogn. 2014;17: 56–72. 10.1017/S1366728912000715

[10] Morgan-Short K, Finger I, Grey S, Ullman MT. Second language processing shows increased native-like neural responses after months of no exposure. PLoS ONE. 2012;7: e32974 10.1371/journal.pone.0032974 22470434 PMC3314650

[11] Morgan-Short K, Sanz C, Steinhauer K, Ullman MT. Second language acquisition of gender agreement in explicit and implicit training conditions: An event-related potential study. Lang Learn. 2010;60: 154–193. 10.1111/j.1467-9922.2009.00554.x 21359123 PMC3044320

[12] Opitz B, Friederici AD. Interactions of the hippocampal system and the prefrontal cortex in learning language-like rules. NeuroImage. 2003;19: 1730–1737. 10.1016/S1053-8119(03)00170-8 12948727

[13] Opitz B, Friederici AD. Brain correlates of language learning: The neuronal dissociation of rule-based versus similarity-based learning. J Neurosci. 2004;24: 8436–8440. 10.1523/JNEUROSCI.2220-04.2004 15456816 PMC6729909

[14] Opitz B, Friederici AD. Neural basis of processing sequential and hierarchical syntactic structures. Hum Brain Mapp. 2007;28: 585–592. 10.1002/hbm.20287 17455365 PMC6871462

[15] Wong PCM, Ettlinger M, Zheng J. Linguistic grammar learning and DRD2-TAQ-IA polymorphism. PLoS ONE. 2013;8: e64983 10.1371/journal.pone.0064983 23741438 PMC3669058

[16] Rossiter MJ. Perceptions of L2 fluency by native and non-native speakers of English. Can Mod Lang Rev. 2009;65: 395–412. 10.3138/cmlr.65.3.395

[17] Weber-Fox CM, Neville HJ. Maturational constraints on functional specializations for language processing: ERP and behavioral evidence in bilingual speakers. J Cogn Neurosci. 1996;8: 231–256. 10.1162/jocn.1996.8.3.231 23968150

[18] Doughty C. Second language instruction does make a difference: Evidence from an empirical study of SL relativization. Stud Second Lang Acquis. 1991;13: 431–469. 10.1017/S0272263100010287

[19] Abrahamsson N, Hyltenstam K. Age of onset and nativelikeness in a second language: Listener perception versus linguistic scrutiny. Lang Learn. 2009;59: 249–306. 10.1111/j.1467-9922.2009.00507.x

[20] Ellis NC. Sequencing in SLA: Phonological memory, chunking, and points of order. Stud Second Lang Acquis. 1996;18: 91–126. 10.1017/S0272263100014698

[21] Baddeley A, Gathercole S, Papagno C. The phonological loop as a language learning device. Psychol Rev. 1998;105: 158–173. 10.1037/0033-295X.105.1.158 9450375

[22] Harrington M, Sawyer M. L2 working memory capacity and L2 reading skill. Stud Second Lang Acquis. 1992;14: 25–38. 10.1017/S0272263100010457

[23] Mackey A, Philp J, Egi T, Fujii A, Tatsumi T. Individual differences in working memory, noticing interactional feedback and L2 development In: Robinson P, editor. Individual differences and instructed language learning. Amsterdam: John Benjamins; 2002 pp. 181–209.

[24] Baddeley A. Working memory and language: An overview. J Comm Dis. 2003;36: 189–208. 10.1016/S0021-9924(03)00019-4 12742667

[25] Daneman M, Carpenter PA. Individual differences in working memory and reading. J Verbal Learn Verbal Behav. 1980;19: 450–466. 10.1016/S0022-5371(80)90312-6

[26] Fedorenko E, Gibson E, Rohde D. The nature of working memory capacity in sentence comprehension: Evidence against domain-specific working memory resources. J Mem Lang. 2006;54: 541–553. 10.1016/j.jml.2005.12.006

[27] Gathercole SE, Service E, Hitch GJ, Adams A-M, Martin AJ. Phonological short-term memory and vocabulary development: Further evidence on the nature of the relationship. App Cogn Psychol. 1999;13: 65–77. 10.1002/(SICI)1099-0720(199902)13:1<65::AID-ACP548>3.0.CO;2-O

[28] Miyake A, Just MA, Carpenter PA. Working memory constraints on the resolution of lexical ambiguity: Maintaining multiple interpretations in neutral contexts. J Mem Lang. 1994;33: 175–202. 10.1006/jmla.1994.1009

[29] Mackey A, Adams R, Stafford C, Winke P. Exploring the relationship between modified output and working memory capacity. Lang Learn. 2010;60: 501–533. 10.1111/j.1467-9922.2010.00565.x

[30] Atkins PWB, Baddeley AD. Working memory and distributed vocabulary learning. Appl Psycholinguist. 1998;19: 537–552. 10.1017/S0142716400010353

[31] Papagno C, Valentine T, Baddeley A. Phonological short-term memory and foreign-language vocabulary learning. J Mem Lang. 1991;30: 331–347. 10.1016/0749-596X(91)90040-Q

[32] Martin KI, Ellis NC. The roles of phonological short-term memory and working memory in L2 grammar and vocabulary learning. Stud Second Lang Acquis. 2012;34: 379–413. 10.1017/S0272263112000125

[33] Ullman MT. A neurocognitive perspective on language: The declarative/procedural model. Nat Rev Neurosci. 2001;2: 717–726. 10.1038/35094573 11584309

[34] Ullman MT. Contributions of memory circuits to language: The declarative/procedural model. Cognition. 2004;92: 231–270. 10.1016/j.cognition.2003.10.008 15037131

[35] Eichenbaum H, Cohen NJ. From conditioning to conscious recollection: Memory systems of the brain Oxford: Oxford University Press; 2001.

[36] Tulving E. What is episodic memory? Curr Dir Psychol Sci. 1993;2: 67–70. 10.1111/1467-8721.ep10770899

[37] Hamrick P. Declarative and procedural memory abilities as individual differences in incidental language learning. Learn Individ Differ. 2015;44: 9–15. 10.1016/j.lindif.2015.10.003

[38] Morgan-Short K, Deng Z, Brill-Schuetz KA, Faretta-Stutenberg M, Wong PCM, Wong FCK. A view of the neural representation of second language syntax through artificial language learning under implicit contexts of exposure. Stud Second Lang Acquis. 2015;37: 383–419.

[39] Ettlinger M, Margulis EH, Wong PCM. Implicit memory in music and language. Front Psychol. 2011;2: 211 10.3389/fpsyg.2011.00211 21927608 PMC3170172

[40] Squire LR, Zola SM. Structure and function of declarative and nondeclarative memory systems. Proc Natl Acad Sci. 1996;93: 13515–13522. 10.1073/pnas.93.24.13515 8942965 PMC33639

[41] Tyler LK, Marslen-Wilson WD, Stamatakis EA. Differentiating lexical form, meaning, and structure in the neural language system. Proc Natl Acad Sci. 2005;102: 8375–8380. 10.1073/pnas.0408213102 15923263 PMC1149400

[42] Ellis R. Principles of instructed language learning. System. 2005;33: 209–224. 10.1016/j.system.2004.12.006

[43] Gibbons P. Scaffolding Robinson P, editor. The Routledge Encyclopedia of Second Language Acquisition. London: Routledge; 2013 pp. 563–4.

[44] Gibbons P. Scaffolding language, scaffolding learning: Teaching second language learners in the mainstream classroom 2nd ed. Portsmouth, NH: Heinemann; 2015.

[45] Hammond J, Gibbons P. What is scaffolding? In: Burns A, de Silva Joyce H, editors. Teachers’ voices 8: Explicitly supporting reading and writing in the classroom. Sydney: National Centre for English Language Teaching and Research, Macquarie University; 2005 pp. 8–16.

[46] Elman JL. Learning and development in neural networks: The importance of starting small. Cognition. 1993;48: 71–99. 10.1016/0010-0277(93)90058-4 8403835

[47] Elman JL. Connectionist models of cognitive development: Where next? Trends Cogn Sci. 2005;9: 111–117. 10.1016/j.tics.2005.01.005 15737819

[48] Hare M, Elman JL. Learning and morphological change. Cognition. 1995;56: 61–98. 10.1016/0010-0277(94)00655-5 7634765

[49] Conway CM, Ellefson MR, Christiansen MH. When less is less and when less is more: Starting small with staged input. In: Alterman R, Kirsh D, editors. Proceedings of the 25th Annual Conference of the Cognitive Science Society. Lawrence Erlbaum Mahwah, NJ; 2003. pp. 270–275.

[50] Kersten AW, Earles JL. Less really is more for adults learning a miniature artificial language. J Mem Lang. 2001;44: 250–273. 10.1006/jmla.2000.2751

[51] Lai J, Poletiek FH. The impact of adjacent-dependencies and staged-input on the learnability of center-embedded hierarchical structures. Cognition. 2011;118: 265–273. 10.1016/j.cognition.2010.11.011 21145537

[52] Lai J, Poletiek FH. How “small” is “starting small” for learning hierarchical centre-embedded structures? J Cogn Psychol. 2013;25: 423–435. 10.1080/20445911.2013.779247

[53] Poletiek F. What in the world makes recursion so easy to learn? A statistical account of the staged input effect on learning a center-embedded structure in artificial grammar learning (AGL). Biolinguistics. 2011;5: 036–042.

[54] Gierut JA. Phonological complexity and language learnability. Am J Speech Lang Pathol. 2007;16: 6–17. 10.1044/1058-0360(2007/003) 17329671 PMC2553565

[55] Kiran S. Complexity in the treatment of naming deficits. Am J Speech Lang Pathol. 2007;16: 18 10.1044/1058-0360(2007/004) 17329672 PMC2731154

[56] Thompson CK, Shapiro LP. Complexity in treatment of syntactic deficits. Am J Speech Lang Pathol. 2007;16: 30 10.1044/1058-0360(2007/005) 17329673 PMC2238729

[57] Gagné RM. The conditions of learning and theory of instruction New York: Holt, Rinehart and Winston; 1985.

[58] Herzog MH, Fahle M. The role of feedback in learning a vernier discrimination task. Vision Res. 1997;37: 2133–2141. 10.1016/S0042-6989(97)00043-6 9327060

[59] Steinhauer K, Grayhack JP. The role of knowledge of results in performance and learning of a voice motor task. J Voice. 2000;14: 137–145. 10.1016/S0892-1997(00)80020-X 10875564

[60] Seger CA. How do the basal ganglia contribute to categorization? Their roles in generalization, response selection, and learning via feedback. Neurosci Biobehav Rev. 2008;32: 265–278. 10.1016/j.neubiorev.2007.07.010 17919725 PMC2376049

[61] Shohamy D, Myers CE, Grossman S, Sage J, Gluck MA, Poldrack RA. Cortico‐striatal contributions to feedback‐based learning: Converging data from neuroimaging and neuropsychology. Brain. 2004;127: 851–859. 10.1093/brain/awh100 15013954

[62] Knowlton BJ, Mangels JA, Squire LR. A neostriatal habit learning system in humans. Science. 1996;273: 1399–1402. 10.1126/science.273.5280.1399 8703077

[63] Metcalfe J, Kornell N, Finn B. Delayed versus immediate feedback in children’s and adults’ vocabulary learning. Mem Cognit. 2009;37: 1077–1087. 10.3758/MC.37.8.1077 19933453

[64] Fyfe ER, DeCaro MS, Rittle-Johnson B. When feedback is cognitively-demanding: The importance of working memory capacity. Instr Sci. 2014;43: 73–91. 10.1007/s11251-014-9323-8

[65] Goo J. Corrective feedback and working memory capacity in interaction-driven L2 learning. Stud Second Lang Acquis. 2012;34: 445–474. 10.1017/S0272263112000149

[66] Goudbeek M, Cutler A, Smits R. Supervised and unsupervised learning of multidimensionally varying non-native speech categories. Speech Commun. 2008;50: 109–125. 10.1016/j.specom.2007.07.003

[67] Russell J, Spada N. The effectiveness of corrective feedback for the acquisition of L2 grammar In: Norris JM, Ortega L, editors. Synthesizing research on language learning and teaching. Amsterdam: John Benjamins; 2006 pp. 133–164.

[68] Berko J. The child’s learning of English morphology. Word. 1958;14: 150–177.

[69] Unterrainer JM, Rahm B, Leonhart R, Ruff CC, Halsband U. The Tower of London: The impact of instructions, cueing, and learning on planning abilities. Cogn Brain Res. 2003;17: 675–683. 10.1016/S0926-6410(03)00191-5 14561454

[70] Phillips LH. The role of memory in the Tower of London task. Memory. 1999;7: 209–231. 10.1080/741944066 10645380

[71] Vicari S, Marotta L, Menghini D, Molinari M, Petrosini L. Implicit learning deficit in children with developmental dyslexia. Neuropsychologia. 2003;41: 108–114. 10.1016/S0028-3932(02)00082-9 12427569

[72] Woodcock RW, McGrew KS, Mather N. Woodcock-Johnson III Tests of cognitive abilities Itasca, IL: Riverside; 2001.

[73] Ferreira VS, Bock K, Wilson MP, Cohen NJ. Memory for syntax despite amnesia. Psychol Sci. 2008;19: 940–946. 10.1111/j.1467-9280.2008.02180.x 18947361 PMC2659624

[74] Christiansen MH, Chater N. Language as shaped by the brain. Behav Brain Sci. 2008;31: 489–509. 10.1017/S0140525X08004998 18826669

[75] Damasio H, Grabowski TJ, Tranel D, Hichwa RD, Damasio AR. A neural basis for lexical retrieval. Nature. 1996;380: 499–505. 10.1038/380499a0 8606767

[76] Tomasello M. Constructing a language: A usage-based theory of language acquisition Cambridge, MA: Harvard University Press; 2003.

[77] Gomez RL. Transfer and complexity in artificial grammar learning. Cogn Psychol. 1997;33: 154–207. 10.1006/cogp.1997.0654 9245469

[78] Kim Y, Payant C, Pearson P. The intersection of task-based interaction, task complexity, and working memory. Stud Second Lang Acquis. 2015;37: 549–581. 10.1017/S0272263114000618

[79] Révész A. Task complexity, focus on form, and second language development. Stud Second Lang Acquis. 2009;31: 437–470. 10.1017/S0272263109090366

[80] Hayes B, Kirchner R, Steriade D, editors. Phonetically based phonology New York: Cambridge University Press; 2004.

[81] Boeckx C. Understanding minimalist syntax: Lessons from locality in long-distance dependencies Malden, MA: Blackwell; 2007.

[82] Murphy GL, Hampton JA, Milovanovic GS. Semantic memory redux: An experimental test of hierarchical category representation. J Mem Lang. 2012;67: 521–539. 10.1016/j.jml.2012.07.005 23243336 PMC3519438

[83] Shohamy D, Adcock RA. Dopamine and adaptive memory. Trends Cogn Sci. 2010;14: 464–472. 10.1016/j.tics.2010.08.002 20829095

[84] Shohamy D, Myers CE, Hopkins RO, Sage J, Gluck MA. Distinct hippocampal and basal ganglia contributions to probabilistic learning and reversal. J Cogn Neurosci. 2008;21: 1820–1832. 10.1162/jocn.2009.21138 18823246

[85] Parbery-Clark A, Skoe E, Lam C, Kraus N. Musician enhancement for speech-in-noise. Ear Hear. 2009;30: 653–661. 10.1097/AUD.0b013e3181b412e9 19734788

[86] Antoniou M, Wong PCM. Poor phonetic perceivers are affected by cognitive load when resolving talker variability. J Acoust Soc Am. 2015;138: 571–574. 10.1121/1.4923362 26328675 PMC4529436

[87] Antoniou M, Wong PCM, Wang S. The effect of intensified language exposure on accommodating talker variability. J Speech Lang Hear Res. 2015;58: 722–727. 10.1044/2015_JSLHR-S-14-0259 25811169 PMC4610280

